# Water Sampling Module for Collecting and Concentrating *Legionella pneumophila* from Low-to-Medium Contaminated Environment

**DOI:** 10.3390/bios11020034

**Published:** 2021-01-27

**Authors:** Khalid Moumanis, Lilian Sirbu, Walid Mohamed Hassen, Eric Frost, Lydston Rodrigues de Carvalho, Pierre Hiernaux, Jan Jerzy Dubowski

**Affiliations:** 1 Laboratory for Quantum Semiconductors and Photon-Based BioNanotechnology, Interdisciplinary Institute for Technological Innovation (3IT), CNRS UMI-3463, Université de Sherbrooke, 3000 boul. de l’Université, Sherbrooke, QC J1K 0A5, Canada; lilian.sirbu@usherbrooke.ca (L.S.); mohamed.walid.hassen@usherbrooke.ca (W.M.H.); eric.frost@usherbrooke.ca (E.F.); 2 Department of Electrical and Computer Engineering, Faculty of Engineering, Université de Sherbrooke, 2500 boul. de l’Université, Sherbrooke, QC J1K 2R1, Canada; 3 Department of Microbiology and Infectiology, Faculty of Medicine and Health Science, Université de Sherbrooke, Sherbrooke, 3001, 12th Avenue North, QC J1K 0A5, Canada; 4 Produits Chimiques Magnus Limitée, 1271, rue Ampère, Boucherville, QC J4B 5Z5, Canada; ldecarvalho@magnus.ca (L.R.d.C.); phiernaux@magnus.ca (P.H.)

**Keywords:** *Legionella pneumophila*, Legionnaires’ disease, water samples, cooling towers, filtration system, concentration factor, recovery rate, bacteria

## Abstract

The detection of water contamination with *Legionella pneumophila* is of critical importance to manufacturers of water processing equipment and public health entities dealing with water networks and distribution systems. Detection methods based on polymerase chain reaction or biosensor technologies require preconcentration steps to achieve attractive sensitivity levels. Preconcentration must also be included in protocols of automated collection of water samples by systems designed for quasi-continuous monitoring of remotely located water reservoirs for the presence of *L. pneumophila*. We designed and characterized a water sampling module for filtration and backwashing intended for analysis of low-to-medium contaminated water, typically with *L. pneumophila* bacteria not exceeding 50 colony-forming units per milliliter. The concentration factors of 10× and 21× were achieved with 0.22 and 0.45 µm filters, respectively, for samples of bacteria prepared in clean saline solutions. However, a 5× concentration factor was achieved with 0.45 µm filters for a heavily contaminated or turbid water typical of some industrial water samples.

## 1. Introduction

Monitoring the presence of pathogenic microorganisms, such as *Legionella pneumophila* (*L. pneumophila*) in industrial and environmental water is of critical importance to the safety and health of the public [1,2]. *Legionella pneumophila* is a Gram-negative bacterium that has been involved in many recent bacterial outbreaks [3,4,5]. It causes Legionnaires’ disease and Pontiac fever, and it is a common source of hospital-acquired pneumonia, especially in immunocompromised patients [6,7]. *Legionella pneumophila* is considered to be a waterborne bacterium [8] that can be found in hot and cold water, cooling towers and air conditioning systems of large buildings, hospitals, nursing homes, hotels and in groundwater [9]. Transmission of *L. pneumophila* to the human host occurs through inhalation of aerosol or contaminated water droplets [10,11]. The difficulty in testing for the presence of this bacterium in environmental sources is related to its association with biofilms and amoebae [12,13,14,15].

Laboratory-based detection of *L. pneumophila* is carried out with the polymerase chain reaction (PCR) technique [16,17], by employing culture growth techniques [18,19,20] or matrix-assisted laser desorption ionization time-of-flight mass spectroscopy (MALDI-TOF) identification [21,22]. The PCR technique provides highly sensitive detection with the limit of detection (LOD) reaching 2 bacteria/mL [23], however, this method suffers from relatively high cost, the necessity to extract bacterial DNA in a small volume while removing PCR inhibitors. Growth of bacteria in a selective culture medium provides the most reliable method for estimation of the number of bacteria. Due to the low concentration of *L. pneumophila* in water sources, a concentration step is often required to deliver sensitive results [2]. This requires an operator to collect water samples from a suspected source and transport them to a laboratory for analysis, which limits the sampling frequency and affects the time to obtain results. To suppress the concentration of *L. pneumophila* to below the critical level, it would be advantageous to perform routine quasi-continuous collection of water samples to be analyzed on the spot, well ahead of the potential outbreak. A possible method of detecting low numbers of bacteria consists of taking the filters from filtration stations and placing them directly on culture media [24]. However, to efficiently fight disease outbreaks, it is important to analyze samples at the source of water [25], especially in view that no scientific evidence has been established for a safe level of *L. pneumophila* contamination [26,27,28,29] and infectious doses were not well defined [30].

Typical detection methods of bacteria are based on the analysis of small volume samples, which may not represent accurately the average concentration of small quantities of pathogens dispersed in a large volume of water [31]. Thus, filtration or centrifugation and concentration of microorganisms from a relatively large volume of water are imperative. Concentrated samples can be analyzed by a wide variety of detection techniques, such as electrochemical immunosensing [32,33] or photoluminescence monitored digital photocorrosion sensing [34,35,36,37].

Filtration operations are classified in terms of the type and size of organisms retained by the filter membrane. The known broad classifications methods: (i) reverse osmosis offering the finest level of filtration available with a filter pore size (FPS) less than 0.001 µm; (ii) nanofiltration (FPS = 0.001 ÷ 0.01 µm); (iii) ultrafiltration (FPS = 0.01 ÷ 0.1 µm) and (iv) microfiltration (FPS = 0.1 ÷ 1 µm) are used to separate bacteria, colloids and larger particulate materials [38,39,40,41]. Filtration operations can use dead-end or crossflow filtration configurations depending on the flow direction relative to the surface of the filter [38]. In dead end filtration, the liquid flow is perpendicular to the filter membrane and materials are retained on the membrane by forming a cake layer that increases resistance to liquid flow with time. In crossflow filtration, the liquid flow is tangential to the filter membrane and the cake layer build-up is limited by the flow against the filter membrane. For instance, Zhang et al. used a ceramic filter-based system (tangential flow filtration) to concentrate *Escherichia coli (E. coli)* from 0.1 to 1 L volumes to 5 to 10 mL, and showed an average recovery rate of around 90% for bacterial concentrations ranging from 10^4^ to 10^6^ CFU/mL [42]. Recently, this group has also speculated that a highly sensitive detection of bacteria at 0.004–0.04 CFU/mL should be possible with a biosensing system capable of detecting *E. coli* at 1–10 CFU/mL [43]. These experiments were carried out for initial concentration of *E. coli* exceeding 10^7^ CFU/mL. Hill et al. investigated the concentration of viruses, bacteria and parasites from an initial 10 L of tap water spiked with 10^6^ CFU, and they demonstrated recovery rates in the range of 49–93% [44]. Furthermore, Polaczky et al. reported the concentration of multiple microbes from 100 L of tap water samples and achieved similar recoveries rate of 51–94% for seed levels between 100 and 1000 CFU [45]. They applied chemical dispersant and Tween 80 to increase microbial recovery efficiencies and performed secondary concentration via centrifugation to reduce sample volume to 5 mL. Other authors have applied various ultrafiltration techniques for the concentration and detection of biological organisms in water [44,46]. For instance, Fry et al. collected large volumes (up to 6000 L) of groundwater samples from different artesian wells concentrated to approximately 50 L. With a second ultrafiltration step, reducing the volume to 0.5 L, they were able to obtain sufficient nucleic acid biomass extracts for further analysis [47]. Winona et al. used a hollow fiber and tangential flow technique to concentrate 2 L samples with viruses from tap, ground and surface water down to 30–50 mL [48]. These authors demonstrated that ultrafiltration could efficiently concentrate viruses over a wide range of water conditions, although low recovery rates were reported due to viral nanoparticles adsorbing excessively to the filter. Magana et al. employed a dead end filtration system for concentrating coliform *E. coli* present in lettuce and spinach wash, and recovering bacterial cells with a buffer solution to produce 400 mL of retentate [49]. They reported 50–58% and 26–63% recovery rates for spiked levels between 1.58 and 1380 CFU/mL and 5 and 2511 CFU/mL of lettuce and spinach wash samples, respectively. Their setup included one pump for filtration and a second syringe pump to perform backflushing; and used one filter that retains not only bacteria, but also large leaf particulates and subsamples, which resulted in approximately up to 10% less filtered volumes than the intended 50 L. Some authors have demonstrated the potential of concentrating various organisms, such as *E. coli* from water matrices by using chemical dispersants and surfactants [44,46]. This was found beneficial for improving recovery rates while recirculating water samples through the original container, although this approach could lead to an increased health hazard to the sample processing personnel.

In this report, we discuss the performance of a water sampling module (WSM) for automated collection of environmental water sources such as cooling tower samples, which has been designed for on-site analysis with a dedicated workstation. The WSM concentration capacity is based on the application of a dead-end filtration cycle followed with backwashing to collect bacterial concentrates. The initial experiments with *E. coli* suspensions allowed us to rapidly determine base parameters of the WSM unit. This was followed by systematic investigation of WSM designed for concentrating bacteria from *L. pneumophila* suspensions. The focus was on collecting samples of water containing *L. pneumophila* at 50 CFU/mL, or below, which is of critical importance to provide early warning of the increased risk of an *L. pneumophila* related health hazard. Preconcentration of *L. pneumophila* present in industrial and environmental water samples at less than 30 CFU/mL is a subject of our ongoing research.

## 2. Materials and Methods

### 2.1. Microorganisms and Materials

Green fluorescent *L. pneumophila* ssp1 (GFP *L. pneumophila* ssp) was kindly provided by Prof. Sébastien Faucher (McGill University, Montréal, Canada), and environmental *L. pneumophila* ssp1 was obtained from Magnus Chemicals (Boucherville, QC, Canada). *Escherichia coli* (*E. coli*) K12 was provided from the bacterial collection of the Biology Department, Faculty of Sciences, Université de Sherbrooke. Buffered charcoal yeast extract (BCYE) agar medium was acquired from Becton, Dickinson and Company (Sparks, MD, USA) and used to grow *L. pneumophila*. Sodium chloride, l-cysteine, Isopropyl-β-d-thiogalactopyranoside (IPTG), chloramphenicol, Casein and phosphate buffered saline (PBS) were purchased from Sigma-Aldrich (Oakville, ON, Canada). Polyvinylidene difluoride (PVDF) filter membranes were purchased from Millipore (Toronto, ON, Canada). When GFP fluorescence was required to aid in counting *L. pneumophila* colonies, IPTG (1 mM) and chloramphenicol (5 µg/mL) were added to the BCYE agar. Deionized (DI) water with an electrical resistivity of 18 MΩ·cm was obtained with a Millipore purification custom system built by Culligan (Québec, QC, Canada). Saline (pristine conditions) was prepared by adding 9 g of NaCl to 1 L of DI water.

GFP *L. pneumophila* ssp1 was cultured on BCYE agar medium supplemented with l-cysteine to promote bacterial growth. Chloramphenicol and IPTG were also incorporated to induce the production of GFP. GFP *L. pneumophila* was used to facilitate colony counting and bacterial identification after growth. Incubation, up to 7 days at 35 °C, was carried out in a commercial biological cabinet Class II (Nu-475-500, Nuaire, Montréal, QC, Canada).

### 2.2. Preparation of Bacteria in Suspension

**Figure 1:**
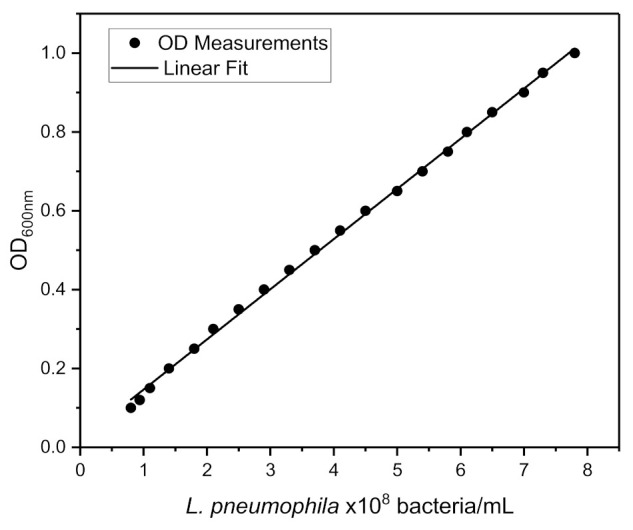
Optical density at 600 nm of live *L. pneumophila* suspensions in 1× PBS. The solid line was obtained by the least-square fitting procedure (R^2^ = 0.99).

A few colonies from *L. pneumophila* cultures were suspended in 1× PBS and their concentration was determined by optical density (OD) measurements at 600 nm using a ThermoFisher spectrophotometer. Figure 1 shows a plot of OD as a function of the concentration of bacteria in suspension. We determined that OD = 0.1 corresponded to 6.4 × 10^7^ of *L. pneumophila*/mL when suspensions were derived from fresh overnight cultures. It can be seen that a linear dependence is observed in the investigated region of concentrations. We employed a similar calibration procedure for establishing concentrations of *E. coli* and we determined that OD = 0.1 corresponded to 8 × 10^7^ *E. coli*/mL.

### 2.3. Water Sampling Module

**Figure 2:**
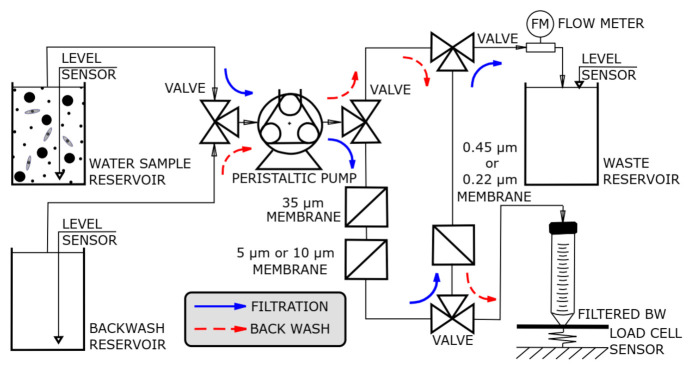
Schematic diagram of water sampling module (WSM) apparatus used for filtering a sample of water and concentrating bacteria (direction of filtration is shown by blue arrows and backwash flow by red arrows).

Figure 2 shows a schematic diagram of the WSM with blue arrows and red arrows indicating the filtration and backwash directions, respectively. The filtration of water samples was performed using PVDF filters with 5, 10 and 35 µm pore diameter membranes designed for removing macroparticles, such as algae, sand and metallic elements, and 0.22 or 0.45 µm pore diameter membranes to collect bacteria. The large pore filters should be useful when collecting water samples from rivers, lakes and industrial water towers. However, these large pore filters are redundant, in most cases, for filtering samples prepared in laboratory conditions. PVDF filter membranes were used considering their hydrophilic properties allowing reduced physisorption of organic material that could alter the backwash efficiency when filtering environmental water samples. The hydrophilicity of such membranes [50] is important for their wettability that provides high flow rates and throughput. Additionally, PVDF filter membranes bind far less protein than other filters [51]. The WSM setup includes four 3-way valves, three reservoirs (water sample, backwash and waste), three level-sensors, a peristaltic pump, a load cell to collect the backwashed (BW) material and a flow meter (FM). The FM allows one to determine the volume of liquids in the reservoirs and, consequently, makes it possible to control the BW process. This device was also employed to complete the moisturizing step designed to condition fresh filters. The level-sensors serve to indicate “empty tanks” with water and BW material, whereas the level-sensor for the waste reservoir serves to detect a “full tank”, in either case resulting in stopping the flow. The WSM electronic block diagram is shown in Figure S1 of Supplementary Materials.

### 2.4. Standard Operating Procedure

During filtration, liquid flows through the membranes and the filtered product was collected in the waste reservoir. After completing the filtration procedure, the valves were switched to allow the BW liquid (1× PBS) to push bacteria accumulated on the microfiltration membrane to a BW tube. The BW volume was controlled by the load cell sensor, while the flow meter monitored the amount of filtered and backwashed water. The WSM apparatus also allowed the addition of chemicals, for instance to create an acidic environment to kill most bacteria except *L. pneumophila*, or for cleaning of the system. The cleaning procedure allows the reuse of filters, which is important for future applications of WSM as a semiautonomous instrument, fully controlled by a microprocessor. The WSM system allows the filtration of water volumes ranging from 0.5 to 10 L and producing 10–15 mL of bacterial concentrates by the BW procedure. The concentration factor (CF) of the system depends on the efficiency of the employed filters and the volume of BW material. Interactive software and data logs give information about the quantity of bacterial solutions produced. The recovery rate (RR) and CF were calculated using the following equations:

$$\mathrm{RR}=\frac{\mathrm{Total}\;\mathrm{number}\;\mathrm{of}\;\mathrm{bacteria}\;\mathrm{in}\;\mathrm{the}\;\mathrm{backwash}\;\mathrm{volume}}{\mathrm{Total}\;\mathrm{number}\;\mathrm{of}\;\mathrm{bacteria}\;\mathrm{in}\;\mathrm{the}\;\mathrm{original}\;\mathrm{volume}}$$

$$\mathrm{CF}=\frac{\mathrm{Average}\;\mathrm{concentration}\;\mathrm{of}\;\mathrm{backwashed}\;\mathrm{bacteria}\;\mathrm{determined}\;\mathrm{by}\;\mathrm{growth}}{\mathrm{Initial}\;\mathrm{concentration}\;\mathrm{of}\;\mathrm{bacteria}\;\mathrm{determined}\;\mathrm{by}\;\mathrm{growth}}$$

### 2.5. WSM Decontamination Procedure

Before each test, the WSM system was decontaminated by 1 h exposure to 1% bleach solution (obtained by 5-fold dilution of a commercial product). Following the decontamination step, the system was washed by flowing 3 L of DI water. Then, 125 mL of a 2% casein solution in DI water was introduced into the system for one hour to passivate the tubing walls, which was designed to prevent physisorption of bacteria [52]. Finally, 1 L of DI water was flown through the system to wash away unbound casein. By activating the BW button, part of the liquid goes to the BW tube, which allows filling of dead volumes. The filtration procedure was carried out each time with a set of new 5, 10 or 35 µm and 0.22 or 0.45 µm filter membranes placed in their dedicated holders. The sterility of WSM was verified with 100 µL of BW material obtained by filtration of 500 mL of DI water. The absence of microbial growth in five Petri dishes inoculated with the product of filtration confirmed the efficiency of sterilization. The sterility of WSM is important for preventing cross-contamination of water samples, even if new filters were used each time when performing filtration and concentration. Different WSM elements could have been contaminated during assemblage and continuous reuse of the system.

### 2.6. Acid Treatment

When isolating *L. pneumophila* from samples of environmental water with potentially high concentrations of different bacteria, acid treatment was used following a procedure recommended by the Centers for Disease Control and Prevention (CDC) [53,54]. The Cooling Tower Water (CTW) product of initial pH 7.4 was brought to pH 2.2 by adding drops of HCl solution (1 M), which required approximately 10–12 min. This was followed by a 5-min incubation at pH 2.2, followed by 10–12 min required to bring the product back to the neutral pH conditions using NaOH solution (1 M). Thus, although the effective time of treatment at pH 2.2 was 5 min, the time to complete the acidification step was 30 min [55]. The pH measurements were carried out with a digital pH meter (Accumet Basic, model AB15, Fisher Scientific, Waltham, MA, USA).

### 2.7. Filtration of Saline Samples Spiked with E. coli

No acid treatment was applied while investigating reference conditions involving *E. coli*. To filter the 10 L volume of saline samples spiked with *E. coli* at 1 × 10^5^, 5 × 10^5^ and 1.2 × 10^6^ CFU/mL, a series of filtration/backwash steps were performed. Typically, a 10 mL volume of BW material was obtained following a 15-min filtration step. The OD values were determined for each 10 mL of collected BW, which was later used to determine bacterial RR and CF.

### 2.8. Dilution and Filtration of Saline Samples Spiked with L. pneumophila at 50 CFU/mL

**Figure 3:**
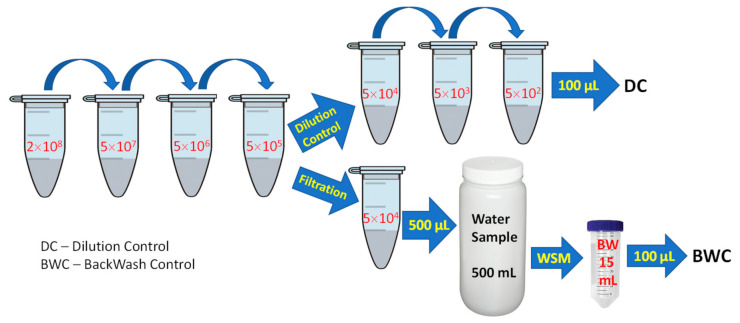
Illustration of the dilution steps employed to prepare water samples spiked with *L. pneumophila* at 50 CFU/mL for dilution and backwashing control (the indicated concentrations are in *L. pneumophila*/mL).

Dilutions were prepared from a freshly prepared suspension of *L. pneumophila* at 2 × 10^8^ CFU/mL as illustrated in Figure 3. The actual number of bacteria per mL in a 500 mL volume of saline was determined by culture and called the dilution control (DC). The average number of bacteria captured by filtration was identified as a backwash control (BWC). The clean saline samples spiked with *L. pneumophila* were tested with and without acid treatment before investigating WSM functionality for filtering industrial water samples inoculated with *L. pneumophila*.

### 2.9. Filtration of Cooling Tower Water (CTW) Samples Spiked with L. pneumophila at 50 CFU/mL

In order to have a sufficient volume of CTW samples, the samples of water collected from various cooling towers, previously confirmed to not contain *L. pneumophila*, were mixed to obtain homogenous material. Environmental *L. pneumophila* ssp1 (control strain) was suspended in 1X PBS to prepare samples nominally with *L. pneumophila* at 50 CFU/mL in a total volume of 500 mL of CTW. The bacteria were filtered using a new 0.45 µm filter, and new 5 µm or coarser filters were used, depending on the quality of sampled water. Prior to spiking CTW samples with *L. pneumophila*, an acid treatment was applied to reduce the contaminating flora.

The CTW material used in this report, identified as M230117, was a mixture of 10 CTW samples originating from different cooling towers operating in the province of Québec, Canada (collected on 11–12 January 2017 and mixed on the 23 January 2017). A fixed 500 mL volume of CTW samples, spiked with *L. pneumophila* was filtered, and the collected BW volume, V_BW,_ was equal to 15 mL.

### 2.10. Growth of the Backwashed Filtrate and Bacteria Quantitation

After completing filtration of either saline or CTW spiked with *L. pneumophila*, a minimum of 5 Petri dishes were inoculated with 100 µL of DC and BWC suspensions of the analyzed material. Bacteria were counted following incubation at 35 °C for 3–7 days. For each test, the RR and CF values were determined based on the knowledge of DC and BWC.

## 3. Results

### 3.1. Recovery Rates and Concentration Factors of Samples Spiked with E. coli

**Figure 4:**
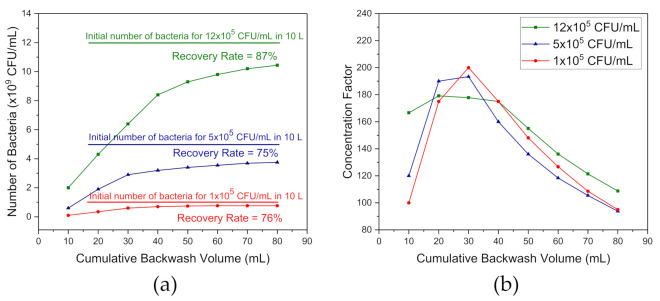
Number of recovered bacteria (**a**) and concentration factor (**b**) versus cumulative backwash volumes for 10 L of saline samples spiked with *E. coli* K12 at 1 × 10^5^, 5 × 10^5^ and 1.2 × 10^6^ CFU/mL.

An initial evaluation of the functioning of the WSM was determined with *E. coli* suspensions at ≥10^5^ CFU/mL in saline. This approach allowed rapid verification of RR and CF data due to the significantly faster dynamics and efficiency of culture of *E. coli* compared to that of *L. pneumophila*. Filtration was carried out with 0.45 and 10 µm filters, and the total volume of bacterial suspensions at 10 L. During the filtration procedure, the flow rates decreased significantly due to fouling and accumulation of bacteria on the 0.45 µm filter. Filtration was completed within 45 min in the case of an initial concentration at 1 × 10^5^ CFU/mL; however, it required 75 min when 5 × 10^5^ and 1.2 × 10^6^ CFU/mL of bacteria were present. Both RR and CF were determined for BW volume ranging from 10 to a total of 80 mL achieved in 8 steps of 10 mL each. Figure 4 shows RR and CF versus cumulative backwash volume for the filtration of 10 L water samples inoculated with *E. coli* K12 at 1 × 10^5^, 5 × 10^5^ and 1.2 × 10^6^ CFU/mL. The results in Figure 4a show that for 1 × 10^5^ CFU/mL, a 76% RR was reached after 4 BW steps. For 5 × 10^5^ and 1.2 × 10^6^ CFU/mL, the RR values achieved with 6 and 8 BW steps were at 75% and 87%, respectively.

With an initial *E. coli* concentration at 1.2 × 10^6^ CFU/mL, assuming that RR = 87%, the total of 10.44 × 10^9^ of bacteria were collected with 8 BW steps, which corresponded to a CF = 108 (1.30 × 10^8^/1.2 × 10^6^), as shown in Figure 4b. With the same number of BW steps, CF = 95 and 94 were obtained for bacterial concentrations at 1 × 10^5^ and 5 × 10^5^ CFU/mL, respectively. These CF values were smaller than the volumetric concentration values of CF_vol_ = 125 that we obtained for 10 L of the initial volume with 8 BW steps, 10 mL each. However, they reflected more accurately the real efficiency of the WSM system. With a single BW step of 30 mL, CF = 200 and 193 were obtained for the initial concentrations of *E. coli* at 1 × 10^5^ and 5 × 10^5^ CFU/mL, respectively. However, CF = 179 was deduced for a total 20 mL BW in the case of 1.2 × 10^6^ CFU/mL. As clearly shown in Figure 4b, a cumulated backwash volume greater than 30 mL causes a dilution of the collected number of bacteria.

### 3.2. Recovery Rates and Concentration Factors of L. pneumophila in Saline Solution

**Figure 5:**
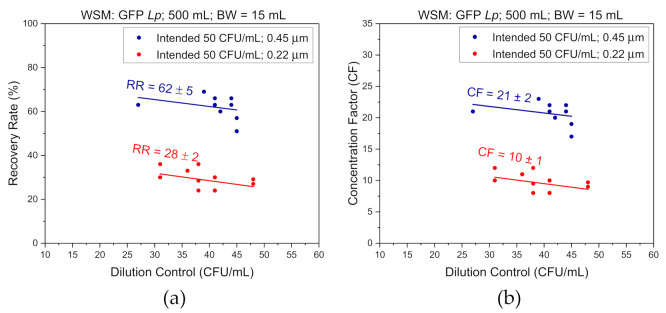
Recovery rate (**a**) and concentration factor (**b**) versus dilution control obtained by filtering saline samples of *L. pneumophila* with 0.22 µm (bottom red solid line) or 0.45 µm (top blue solid line) filters.

Figure 5 shows a summary of the results obtained for filtration runs carried out with either 0.22 or 0.45 µm retention filters for bacterial suspensions at <50 CFU/mL in 500 mL of saline. A 1-step 15 mL BW procedure was employed in this case. The RR and CF values shown in Figure 5a,b, respectively, were determined based on culture evaluation. It can be seen that in the case of the 0.22 µm membrane, the DC values shown in Figure 5 varied between 31 and 48 CFU/mL. The RR and CF values were calculated by dividing BWC × 15 mL by their respective DC × 500 mL values, and BWC by their respective DC values, respectively. The results for the 0.22 µm filter show that RR is varying between 24 and 36%, averaging, RR = 28% ± 2%. This corresponds to CF varying between 8 and 12, averaging CF = 10 ± 1. For the tests involving filtration with the 0.45 µm filter, the estimated DC values are in the range between 27 and 45 CFU/mL. In this case, RR = 62% ± 5% and CF = 21 ± 2, i.e., they are two times greater than those values obtained with the 0.22 µm filter.

### 3.3. Recovery Rates and Concentration Factors of L. pneumophila in Saline Solution Following Acid Treatment

The results obtained for filtration runs carried out with 0.45 µm retention filters are summarized in Table 1. It can be seen that the verified DC values range between 30 and 45 CFU/mL. Furthermore, the CF values of 19–29 (CF_av_ = 22 ± 3) obtained with the acidification step are comparable to those obtained without acidification as reported in Figure 5. Thus, the introduction of the acidification step, while important for the elimination of non-*L. pneumophila* bacteria, did not affect significantly the CF value. It is worthwhile to notice that the average RR_av_ = 65 ± 10 were slightly greater than the RR values deduced with 0.45 filters applied in the non-acid treatment experiments reported in Figure 5.

### 3.4. Recovery Rates and Concentration Factors of L. pneumophila in Acid Treated Industrial Water

The performance of WSM was investigated by concentrating live *L. pneumophila* from CTW samples spiked with *L. pneumophila* at intended 50 CFU/mL in a total volume of 500 mL. Before beginning the filtration process, the sampled suspension of bacteria was acid processed as described in Section 2. Table 2 shows BWC, RR and CF data for *L. pneumophila* concentrations (DC) in the range of 37–56 CFU/mL obtained with the 0.45 µm filter. It can be seen that an average CF = 5 was obtained for the investigated bacterial suspensions of 37–56 CFU/mL. This is approximately 4 times less efficient concentration than that demonstrated for the relatively pristine conditions reported in Figure 5 and Table 1.

## 4. Discussion

The experiments carried out for *E. coli* revealed RR as high as 87% for bacterial suspension at 1.2 × 10^6^ CFU/mL, and 75% for suspensions at (1–5) × 10^5^ CFU/mL. These RR are within the range of 68–93% reported in the literature [42,43,44,56]. For instance, Zhang et al. [42] reported that filtration of 500 mL of water spiked with *E. coli* at 4 × 10^5^ CFU/mL gave RR in the range of 72–93%. However, attaining the highest RR required complicated procedures employing, for instance, two pumps to change the flow direction to reduce fouling and avoid excessive adsorption of bacteria on the employed 0.14 µm pore size filters [42]. The use of small pore size filters for processing of industrial water samples would require prefiltration to remove macrocontaminants, but this would also increase the risk of partial elimination of bacteria from the concentration procedure due to binding or capture of bacteria to macrocontaminants. The use of the tangential flow technique has the potential to alleviate problems related to blocking dead-end filters by heavily contaminated water samples. However, the reported RR slightly exceeding 50% for samples with viruses, bacteria and parasites [31], and inconsistent results concerning filtration of different microorganisms, such as *Bacillus anthracis* and bacteriophages MS2 [57], suggest that the dynamics of filter cake formation and changes in filtration pressure, as investigated for aqueous colloids [58], could play an important role in efficient filtration of bacteria and accurate determination of RR and CF values.

To collect filtrates from 10 L samples of saline spiked with 1 × 10^5^ CFU/mL of *E. coli*, we employed three backwashes of 10 mL each. The filtration procedure in this case took approximately 45 min. However, up to 75 min were required to complete the filtration of saline spiked with (5–12) × 10^5^ CFU/mL of *E. coli*. The increase in the filtration time of more concentrated bacterial suspensions was also associated with increased pressure in the WSM unit, which could also lead to damaging of some bacteria captured by the filter and, consequently, result in an underestimation of the bacterial count based on growth. The CF of 200, 193 and 178 were obtained for 1 × 10^5^, 5 × 10^5^ and 1.2 × 10^6^ CFU/mL suspensions, respectively, based on the 3-step backwashing procedure (BW = 30 mL). The increased pressure during filtration could led to damaging of some bacteria and, consequently, produce lower CF due to a reduced cultivation-based detection. Furthermore, reducing the accumulated BW volume could prevent the underestimation of recovered bacteria and, consequently, lead to more accurate RR and CF values.

The collection of filtrates from saline spiked with *L. pneumophila* at ≤50 CFU/mL was obtained with one 15 mL backwash step in order to minimize the dilution of a final product. In comparison to the filtration with 0.45 µm membranes, the lower values of RR and CF obtained with 0.22 µm membranes are likely related to the reduced efficiency of eluting bacteria from a small pore size structure of membranes. These results are consistent with the results of De Giglio et al. [24] who reported recovery rates of *L. pneumophila* at 65% and 15% for 0.45 and 0.2 µm membranes, respectively.

Saline samples that provide appropriate physiological conditions do not inhibit *L. pneumophila* recovery as it was reported that such solutions can be considered for *L. pneumophila* suspensions [10]. Normal environmental conditions might be closer to distilled water, but river water has some salts in it [59], and CTW will often have salts and sometimes even high levels of salts [60]. The quality of processed water influenced significantly the ability to carry out uninterrupted filtration. Most of the microcontaminants could be removed from CTW samples with 5 µm filters. In the case of amoebae carrying *L. pneumophila*, the use of a 10 µm filter membrane would be sufficient to eliminate amoebae from the stream of a filtered suspension. The injection of a weak aqueous solution of sodium dodecyl sulfonate (0.5%) would lyse the amoeba wall [61] leading to retention of the released *L. pneumophila* by the 0.45 µm filter. The large pore size (up to 35 µm) filters, although not expected to retain bacteria, could immobilize bacteria that bound to the microcontaminants or were trapped by precipitates. In addition to the loss of bacteria, this also resulted in an increase of the filtration time between 15 and 25 min for some of the samples investigated in our experiments, with several interruption steps applied to unclog the filters. Furthermore, in one case, the presence of *L. pneumophila* was detected on the surface of a 35 µm filter mounted in a Petri dish with *L. pneumophila* culture medium, which illustrates the possibility of underestimating bacterial concentrations in heavily contaminated samples.

In the case of CTW samples, WSM-based filtration concentrated *L. pneumophila* with RR = 12–18% and CF = 4–6 (see Table 2). The ~4 times reduced CF in comparison to that obtained for saline samples is probably related to the increased presence of a variety of contaminants in CTW samples. It should be noticed that a comparable concentration efficiency (CF = 4) of *L. pneumophila* in CTW was also reported by Leskinen et al. [62]. We argue that with a CF = 5 produced by WSM employed for processing CTW with *L. pneumophila*, and assisted by the chemotaxis effect [63], an LOD approaching 10 CFU/mL could be obtained reproducibly with a workstation designed for automated biosensing. An LOD of 4 was reported recently for the detection of *L. pneumophila* in freshwater environments, which are less turbid sources, by using a combination of sample concentration, immunoassay detection and measurement by chronoamperometry [64].

The WSM unit was not considered as a substitute for traditional culture, qPCR methods or other molecular biosensing technologies. The backwashed material collected by WSM could be used for further analysis, with either these well-established techniques or with other biosensors attempting to achieve an improved LOD. Clearly, WSM with reusable filters could replace vacuum filtration systems, while interfaced with a biosensing platform capable of a rapid analysis of the backwash material. This approach could represent a paradigm shift in advanced methods of controlling outbreaks of diseases induced by the proliferation of pathogenic bacteria in water and especially cooling tower water.

## 5. Conclusions

In summary, we developed a water sampling module for automated collection of samples of water with low levels of *L. pneumophila* (<50 CFU/mL). The experiments were carried out by backwashing bacteria from dead-end 0.22 and 0.45 µm filters, with bacteria being enumerated at each step by the culture. An initial verification of the performance of the module was performed with pristine suspensions of *E. coli* in saline at 10^5^–10^6^ CFU/mL and revealed recovery rates of 75–87% and concentration factors of 200. These contrasted with concentration factors of 20 and recovery rates of 50–88% for *L. pneumophila* in saline at 30–45 CFU/mL. *Legionella pneumophila* suspensions at similar concentrations in cooling tower water revealed 4-fold reduced concentration factors, which highlights the challenge typical of extracting bacteria from heavily contaminated environments. Furthermore, in comparison with 0.45 µm filters, experiments with 0.22 µm filters yielded slightly lower concentrations of bacteria related to the less efficient backwash process from fine filters. The investigated water sampling module has been designed to be an integral part of a workstation for automated monitoring of water reservoirs (e.g., cooling tower water) for the presence of *L. pneumophila*. With a biosensor exhibiting an LOD at 5 × 10^2^ CFU/mL, our results suggest the feasibility of automated monitoring of industrial water reservoirs for the presence of *L. pneumophila* with LOD at 100 CFU/mL, while implementing the chemotaxis effect should allow automated detection of these bacteria with an LOD approaching 10 CFU/mL.

## Figures and Tables

**Table 1:** Backwashed concentration (BWC), recovery rate (RR) and concentration factor (CF) obtained with a 0.45 µm filter for the indicated concentrations (DC) of *L. pneumophila* in saline samples.

Average DC (CFU/mL)	Average BWC (CFU/mL)	RR (%) (BWC × 15 mL/DC × 500 mL)	CF (BWC/DC)
30	875	88	29
44	1100	75	25
39	884	68	23
41	870	64	21
45	870	58	19
45	750	50	17
42	830	59	20
44	960	65	22
44	910	62	21
41	873	64	21

**Table 2:** Backwashed concentration (BWC), recovery rate (RR) and concentration factor (CF) obtained with a 0.45 µm filter for the indicated concentrations (DC) of *L. pneumophila* in cooling tower water samples.

Average DC (CFU/mL)	Average BWC (CFU/mL)	RR (%) (BWC × 15 mL/DC × 500 mL)	CF (BWC/DC)
56	280	15	5
37	185	15	5
37	149	12	4
44	262	18	6
45	220	15	5

## Data Availability

Not applicable.

## Supplementary Materials

The following are available online at https://www.mdpi.com/2079-6374/11/2/34/s1, Figure S1: Electronic block diagram of a Water Sampling Module.
