# Supplementary material for: Water Sampling Module for Collecting and Concentrating Legionella pneumophila from Low-to-Medium Contaminated Environment
Source: Biosensors (Basel). 2021 Jan 27;11(2):34. doi: 10.3390/bios11020034 (PMC7910891; doi:10.3390/bios11020034)
Supplement: Supplementary file 1 [file biosensors-11-00034-s001.pdf]

biosensors

MDPI

Supplementary Information

Water Sampling Module for Collecting and Concentrating *Legionella pneumophila* from Low-to-Medium Contaminated Environment

Khalid Moumanis [1,*], Lilian Sirbu [1], Mohamed Walid Hassen [1], Eric Frost [1,2], Lydston Rodrigues de Carvalho [3], Pierre Hiernaux [3] and Jan Jerzy Dubowski [1,*]

[1] Interdisciplinary Institute for Technological Innovation (3IT), Laboratory for Quantum Semiconductors and Photon-based BioNanotechnology, CNRS UMI-3463, Department of Electrical and Computer Engineering, Faculty of Engineering, Université de Sherbrooke, 3000 boul. de l'Université, Sherbrooke, Québec J1K 0A5, Canada; lilian.sirbu@usherbrooke.ca (L.S.); mohamed.walid.hassen@usherbrooke.ca (M.W.H.)

[2] Department of Microbiology and Infectiology, Faculty of Medicine and Health Science, Université de Sherbrooke, Sherbrooke, 3001, 12th Avenue North, Québec J1K 0A5, Canada; eric.frost@usherbrooke.ca

[3] Produits Chimiques Magnus Limitée, 1271, rue Ampère, Boucherville, Québec J4B 5Z5, Canada; ldecarvalho@magnus.ca (L.R.C.); phiernaux@magnus.ca (P.H.)

* Correspondence: khalid.moumanis@usherbrooke.ca (K.M.); jan.j.dubowski@usherbrooke.ca (J.J.D.)

The electronic block diagram of the WSM system, as presented in Figure S1, is based on an ATmega32 microcontroller unit (MCU). The MCU custom board ensures a simple and cost attractive platform for prototype solutions. The compact design provides connection of all pins of the MCU to the user. The separate circuits are designed to control the high current spikes with isolator components, and drive digital buffers for water level sensors, amplifiers for load cell, a circuit for peristaltic pump speed control, and a serial to USB adapter CP2102. The main board is equipped with programming ports JTAG and ISP, a keyboard, and an LCD screen. The MCU is driven by a 16 MHz crystal on board and 32.768 Hz for controlling with 1 Hz time base (clock). The valve circuit is operated by a power supply designed to provide a 2-step power source for the electronic control in the range of 3.2-12V, which helps to avoid overheating. The 3-way valves are operated by applying DC and 4/12V controller. A digital buffer interface, based on simple logic elements, was applied with the opto-coupler connection implemented as well. To ensure sufficient current for valves, the opto-couplers are connected to a Darlington transistor TIP125. The level sensors have a digital buffer with integrated opto-couplers.

The signal from a magnetic switch (or reed switch) is connected to the MCU input through the pull-up resistor to the power supply of 5 V. The load cell is formed with a Wheatstone bridge circuit, where all resistors may vary and the circuit is known as a full-bridge type 1. Such bridge is formed by four strain-gauge elements that are compensated for temperature and for lead resistance. Load cell (Strain Gauge/piezoelectric) is supplied with 10 V and connected to a non-inverting operational amplifier. The maximal voltage deviation for load cell of 100 mL volume does not exceed 5 mV.

The speed of the peristaltic pump is controlled by converting ATmega32 pulse wave modulation (PWM) digital signal to analog signal to increase the bias from 5V to 10V. For that, it is necessary to use a non-inverting amplifier and a low-pass filter (integrator) that remove any digital components from the signal.

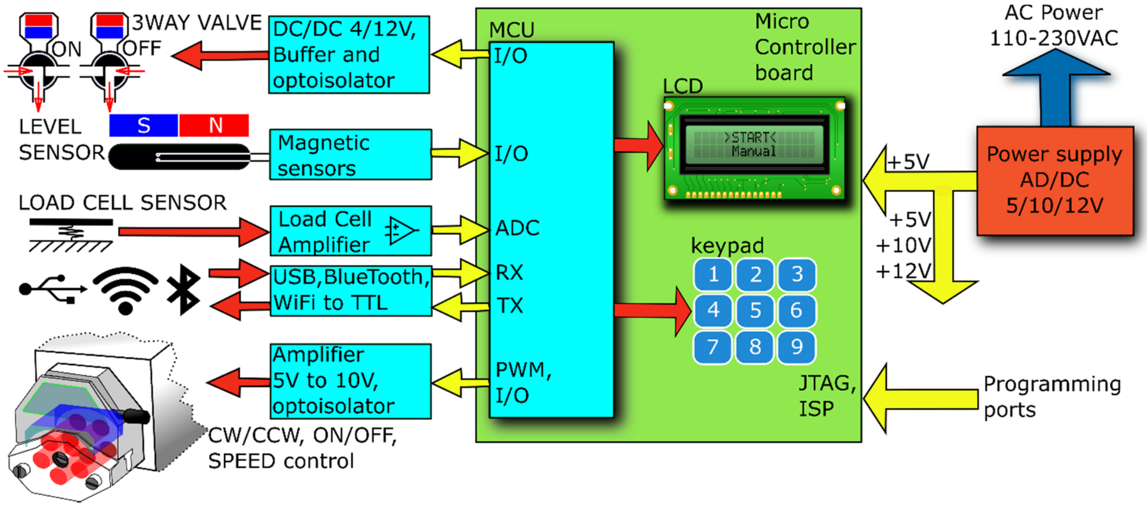

Figure S1. Electronic block diagram of a Water Sampling Module.

© 2021 by the authors. Submitted for possible open access publication under the terms and conditions of the Creative Commons Attribution (CC BY) license (http://creativecommons.org/licenses/by/4.0/).
